# Dieting for Health

**Published:** 1859-01

**Authors:** 


### DIETING FOR HEALTH

Has sent many an one to the grave, and will send many more, because it is done injudiciously or ignorantly. One man omits his dinner by a herculean effort, and thinking he has accomplished wonders, expects wonderful results, but by the time supper is ready he feels as hungry as a dog, and eats like one, fast, furious and long. Next day he is worse, and "don't believe in dieting" for the remainder of life.

Others set out to starve themselves into health, until the system is reduced so low that it has no power of resuscitation, and the man dies.

To diet wisely, does not imply a total abstinence from all food, but the taking of just enough, or of a quality adapted to the nature of the case. Loose bowels weaken very rapidly—total abstinence from all food increases the debility. In this case food should be taken, which while it tends to arrest the disease, imparts nutriment and strength to the system. In this case, rest on a bed, and eating boiled rice, after it has been parched like coffee, will cure three cases out of four of common diarrhœa in a day or two.

Others think that in order to diet effectually, it is all important to do without meat, but allow themselves the widest liberty in all else. But in many cases, in dyspeptic conditions of the system particularly, the course ought to be reversed, because meat is converted into nutriment with the expenditure of less stomach power than vegetables, while a given amount of work does three times as much good, gives three times as much nutriment and strength as vegetable food would.

These "principles" merit consideration, and are more fully stated in our new dollar book on "Health and Disease."

